# Genetic and phylogenetic analysis of the outer capsid protein genes of Indian isolates of bluetongue virus serotype-16

**DOI:** 10.14202/vetworld.2018.1025-1029

**Published:** 2018-08-01

**Authors:** Arpit Saxena, Sanchay K. Biswas, Karam Chand, Jishnu Naskar, Ankita Chauhan, Gulam Mohd, Neha Tewari, Kurat-ul-Ain, Muthannan A. Ramakrishnan, Awadh Bihari Pandey

**Affiliations:** 1 Division of Virology, Indian Veterinary Research Institute (IVRI) Mukteswar, Nainital - 263 138, Uttarakhand, India; 2 Department of Molecular and Cellular Engineering, Sam Higginbottom University of Agriculture Technology and Sciences (SHUATS), Allahabad - 211 007, Uttar Pradesh, India; 3 Division of Biological Standardization, Indian Veterinary Research Institute (IVRI) Izatnagar, Bareilly - 243 122, Uttar Pradesh, India

**Keywords:** bluetongue virus, phylogenetic analysis, *VP2* gene, *VP5* gene

## Abstract

**Aim:**

The aim of the study was to characterize bluetongue virus serotype 16 (BTV-16), recently isolated from different states of India. The evolutionary relationship of newly isolated BTV-16 and previously reported Indian and global BTV-16 isolates were compared using molecular analysis.

**Materials and Methods:**

In the present study, five (n=5) BTV-16 isolates were used to amplify gene segment-2 and segment-6 encoding the outer capsid proteins *VP2* and *VP5*, respectively. The amplified products were purified and sequenced by the Sanger sequencing method. The phylogenetic relationship and nucleotide identity of all five BTV-16 isolates were compared with previously reported Indian and global BTV-16 isolates. Nucleotide sequence data were aligned using the CLUSTAL W algorithm implemented in the MegAlign of DNASTAR program package (MegAlign 5.00, DNASTAR Inc., Madison, USA). Phylogenetic analyses were carried out using MEGA version 6.0 software with the best nucleotide substitution model.

**Results:**

Phylogenetic analysis based on the *VP2* and *VP5* encoding genes, segregates Indian BTV-16 isolates in a distinct cluster with proximity to the Eastern topotype. Indian isolates make a monophyletic cluster with Eastern topotypes with Western topotype BTV-16 (BTV-16/NIG/AJ586694) occupying a separate cluster. Indian isolates were found to share 91.5%-97.5% and 96.5%-98.9% identity at the nucleotide and deduced amino acid (aa) level, respectively, to the global BTV-16 isolates. There is a high degree of variation with the Nigerian isolate with 27.0-27.7% and 26.0-26.9% at the nucleotide and aa sequence level, respectively. These data suggest that Indian BTV-16 isolates might have evolved separately within the Eastern BTV topotype.

**Conclusion:**

Phylogenetic analyses and nucleotide identity of BTV-16 isolates at the *VP2* and *VP5* gene encoded level indicate that isolates used in the present study might have evolved from a common Eastern topotype ancestor. The data presented in this study will be helpful for future selection of reference strains in a serological and molecular epidemiology study.

## Introduction

Bluetongue is the most common, economically important vector-borne viral disease of domestic and wild ruminants [1]. The causative agent of the disease is bluetongue virus (BTV) which belongs to the family Reoviridae within the genus Orbivirus. BTV Infection is commonly seen in sheep and goats. The disease causes economic losses devastating the economic condition of poor farmers and the small ruminant industry, due to often high morbidity, mortality, reproductive failure, reduction in milk yield, and weight loss [2]. BTV is a non-enveloped icosahedral virus particle covered with three structural layers. The complete genome of the virus has 10 double-stranded RNA (dsRNA) segments with seven structural (*VP1*-*VP7*) and four nonstructural proteins (*NS1, NS2, NS3/NS3a*, and *NS4*) [3,4]. Virus proteins *VP1*, *VP3*, *VP4*, *VP6*, and *VP7* represent inner coat structural proteins in addition to core viral particle [5,6]. These core virus proteins are relatively conserved, and also antigenically cross-reactive between different BTV serotypes and strains. The core is surrounded by *VP2* and *VP5* outer capsid proteins encoded by segment-2 and segment-6 of the BTV genome, respectively. The outer capsid protein *VP2* is assembled over a *VP5* scaffold; both of these associates to, determine the serotype of virus and induce a host protective immune response by eliciting neutralizing antibodies [7]. Viral protein *VP5* assists in virus neutralization and enhances the neutralization activity of *VP2* protein [8]. It has been reported that *VP2* and *VP5* genes show genetic reassortment within same and different serotypes [9].

Genetic characterization of different BTV strains has revealed the existence of distinct eastern and western topotypes associated with specific geographical origins of the virus [10]. Constantly, new BTV serotypes are being introduced globally, due to the rapid evolutionary changes in the BTV genome through reassortment, mutations, and intragenic recombination [11]. A total of at least 27 serotypes of BTV have been reported worldwide, whereas 23 distinct BTV serotypes have been reported from India based on serology and virus isolation [12]. Sequence data of the aforesaid two genes of the BTV strains could be used to find out possible variation(s) in them and utilized for vaccine matching. Further, this analysis may provide data for the future epidemiological study of the BTV strains.

The present study was carried out to characterize and identify the phylogenetic relationship of Indian BTV-16 virus isolates from different parts of the country. The segment-2 and segment-6 (*VP2* and *VP5*) of five Indian isolates were sequenced, and phylogenetic analysis was carried out and compared to previously reported Indian and global isolates.

## Materials and Methods

### Ethical approval

Ethical approval was not required in this study. The study contains only molecular related research work. Live animals were not used in this work.

### Cells and virus

A total of five BTV-16 isolates (Table-1) used in the present study were originally isolated in the BHK-21 cells from the blood samples [13]. The isolated viruses were maintained above five passage level at the virus repository, Division of Virology, ICAR-Indian Veterinary Research Institute, Mukteswar. The viruses were revived and grown on BHK-21 cells; the cultures were harvested after 48 h post-infection when more than 80% of the cells showed characteristic cytopathic effects. The infected cultures were processed immediately or stored at 4°C before extraction of viral RNA.

**Table-1:** Details of BTV16 isolates used in the present study.

Isolate ID	Source	Species	Accession no. VP2	Accession no. VP5	Country
GNT7/07/IND	Blood	Sheep	MG710529	MG710532	India
MUK1/12/IND	Blood	Goat	KY934050	MG710533	India
PDP2/13/IND	Blood	Goat	KY934051	MG710534	India
SRL69/IND	Blood	Sheep	MG710530	MG710535	India
KAR50/IND	Blood	Sheep	MG710531	MG710536	India

BTV16=Bluetongue virus serotype 16

### Extraction of viral dsRNA

Viral dsRNA was extracted from the infected cultures by Trizol extraction followed by sequential precipitation with lithium chloride. Briefly, the infected culture lysates were centrifuged at 1200× *g* for 15 min at 4°C to harvest the viruses along with the cellular debris. The pellet was resuspended in Tris-EDTA buffer (pH 7.4) and subsequently digested with a lysis buffer containing 0.5% SDS, 300mM sodium acetate (pH4.5), and proteinase-K at 56°C for 30 min. Total RNA was extracted from the lysate by Trizol extraction following the manufacturer’s protocol. Viral dsRNA was subsequently purified by lithium chloride precipitation as described earlier [14].

### Designing of oligonucleotide primers and amplification of target sequences

The oligonucleotide primer pairs for amplification of the *VP2* and *VP5* genes were designed from the sequences available at the public database (GenBank). The primer pairs were designed using a software program (PrimerSelect 5.00, DNA STAR) and designed to amplify the complete coding sequence in multiple overlapping fragments (Table-2). Complementary DNA was prepared from the dsRNA preparation by reverse transcription using random hexamer primers as described earlier [15]. The target sequences were amplified by PCR using the Phusion High-Fidelity DNA Polymerase Master Mix (Thermofisher Scientific, USA) under the following conditions: Initial denaturation at 95°C for 5 min, then denaturing at 95°C for 30 s, annealing at 48°C for 30 s, and extension at 72°C for 30 s for 35 cycles. The amplified products were checked in 1% agarose gel, and the specific amplicons were purified from the gel using QIAquick Gel Extraction Kit (Qiagen, USA) for Sanger sequencing using specific primers or primer walking.

**Table-2:** List of oligonucleotide primers used for PCR amplification of BTV16 segment2 and segment6

Primer ID	Primer Sequence (5’—3’)	Location
BTV16VP2_F1	GTTAGCCTAGAGATGGAGG	119
BTV16VP2_R1	CACAATGATTCCCCGTATGA	13601341
BTV16VP2_F2	CACATGCGTTTAGGCGATAA	688707
BTV16VP2_R2	CTCACCAAAAGTTGGGAAGA	21832164
BTV16VP2_F3	ACGAATCATACGGGGAATCA	13361355
BTV16VP2_R3	AAGTGTAAACGCAATCCCTGTCA	29292907
BTV16VP5_F1	CCCTACGATTGCGGAAGAT	1230
BTV16VP5_R3	TAAGTGTAAGTCCCGAGATT	16171636

BTV16=Bluetongue virus serotype 16,PCR=Polymerase chain reaction

### Phylogenetic analysis of the sequence data

The nucleotide sequence data of *VP2* and *VP5* gene generated in multiple overlapping fragments were assembled to obtain the complete coding sequence of the genes. The identity of the sequences was confirmed by BLASTN (http://www.ncbi.nlm.nih.gov/) analysis. Nucleotide sequences were aligned with other published sequences using the CLUSTAL W algorithm [16] implemented in the MegAlign of DNASTAR program package (DNASTAR Inc., Madison, USA). Phylogenetic analyses were carried out using MEGA version 6.0 software [17]. The best nucleotide substitution model was selected from the model selection program implemented in MEGA version 6.0 software using the Bayesian Information Criterion. The evolutionary history was inferred using the Maximum Likelihood (ML) method and the bootstrap consensus tree inferred from 500 replicates [18].

## Results

### Analysis of nucleotide sequence data

Analysis by BLASTN confirmed the assembled sequences as the coding region of the cognate gene of BTV-16. The complete coding region of *VP2* was found to be 2880 nucleotides long (including stop codon) encoding for 960 aa. The stop codon TAA was found to be conserved on *VP2* gene in all the virus isolates. A higher percentage of identity was observed among Indian BTV-16 isolates at both nucleic acid and deduced aa sequence levels. Indian isolates were found to share 98.0-99.8% identity at both nucleotides and deduced aa sequence levels. In contrast, the isolates were found to share 91.5-97.5% and 96.5-98.9% identity at the nucleotide and deduced aa level, respectively, to the global BTV-16 strains used in the analyses.

The complete coding region of *VP5* gene of BTV16 isolates used in the present study carries 1581 nucleotides including the stop codon. Consequential *VP5* translated DNA has 526 aa and stop codon TGA was found conserved in all the sequences. Comparison of complete *VP5* encoding genes of the present study with other Indian isolates showed 99.1-99.9% identity at both nucleotides and deduced aa sequence levels. Percent identity of *VP5* gene among Indian and global BTV-16 isolates was found to be 96.4-98.0% for both nucleotide and aa levels.

### Phylogenetic analysis based on VP2 and VP5 gene

**Figure-1:**
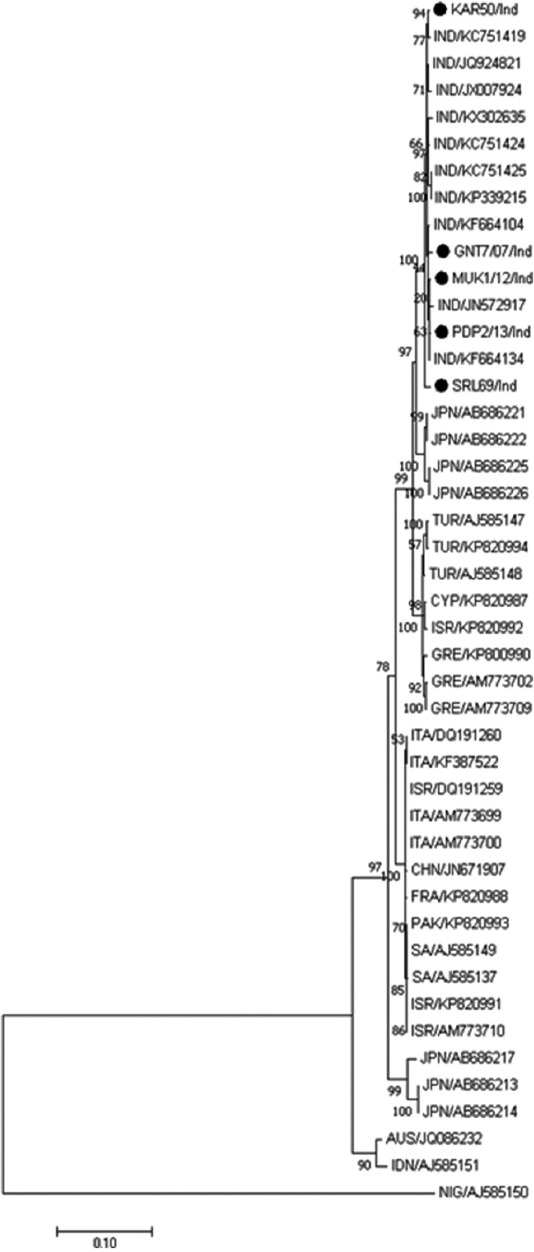
Maximum likelihood tree depicting phylogenetic relationship among BTV-16 isolates from India and other countries in their *VP2* nucleotide sequence. Bootstrap consensus was inferred from 500 replicates. Bluetongue virus serotype 16 isolates from different geographical regions make two major clusters of eastern and western topotype.

The best nucleotide substitution model for phylogenetic analysis of the *VP2* gene was found to be Tamura-Nei with discrete gamma distribution rates across sites (TN93+G). Indian BTV-16 isolates were segregated to a distinct cluster at proximity to the viruses recovered from Turkey, Cyprus, and Japan. Australian BTV-16 isolates were found to be more distantly related with the strains circulating in India. The SRL69/Ind was segregated alone from the major cluster of Indian BTV-16 isolates (Figure-1).

**Figure-2:**
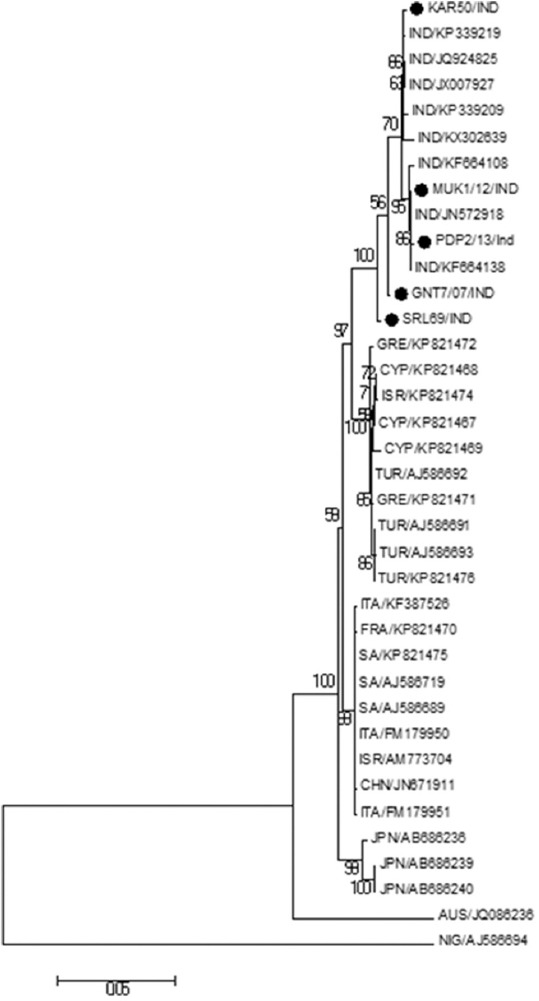
Maximum likelihood tree depicting phylogenetic relationship among Bluetongue virus serotype 16 (BTV-16) isolates from India and other countries in their *VP5* nucleotide sequence. Bootstrap consensus was inferred from 500 replicates. BTV-16 isolates from different geographical regions make two major clusters of eastern and western topotype.

A similar pattern of segregation was also observed in the phylogenetic analysis based on *VP5* gene of global BTV-16 isolates. The ML tree constructed with Tamura-Nei with a fraction of sites assumed to be invariable (TN93+I) as the best suitable model. Phylogenetic analysis segregated Indian BTV-16 isolates in separate cluster distant from the isolates reported from Japan and Australia. The SRL69/Ind isolate was segregated alone from the major cluster of Indian BTV-16 isolates as observed in the ML tree based on *VP5* gene (Figure-2).

## Discussion

In the present study, *VP2* and *VP5* encoding genes were analyzed to characterize Indian BTV-16 isolates and to understand the phylogenetic relationship of the viruses. Although segment-2 is the most variable among the BTV genome segments, analysis of the complete coding region of segment-2 of Indian BTV-16 isolates revealed an extremely high level of nucleotide and deduced aa sequence identity (98.0-99.8%). Variation in the outer capsid protein genes (*VP2* and *VP5*) to different extents is reported among the isolates of a particular serotype in different topotypes [9]. A high percentage of sequence identity was also observed between Indian, and most of the other global BTV-16 isolates except NIG/AJ586694 reported from Nigeria [19]. A new BTV 16 isolate from Andhra Pradesh was found to be very close to other Indian BTV 16 isolates at the nucleotide sequence identity of segment 6 [20]. In this study, a higher percentage of similarity among most of the globally circulated BTV-16 isolates at *VP2* gene nucleotide and deduced aa sequence levels indicate that the Eastern isolates/segment might have evolved from a common ancestor. This is again supported by the percentage identity of the *VP5* gene of the Global BTV16 isolates where 96.4-98.0% similarity was observed at both nucleotides and deduced aa sequence levels.

Indian BTV 16 isolate has been reported previously as having undergone reassortment of segment 5 with more than 88.5% nucleotide sequence identity with the Western topotype [21]. The same isolate VJW64/08/IND has been found to be closely related to the Eastern strain only as reported earlier on the basis of segment-2 (JN572917) and segment-6 (JN572918) nucleotide sequence comparison [9]. Within a single BTV serotype, *VP2* and *VP5* genes show variations that reflect the geographic origins of the virus isolates or genome segment that clearly identify distinct topotypes of the virus [22]. ML tree based on *VP2* gene segregated Indian BTV-16 isolates at proximity to the viruses recovered from Turkey, Cyprus, and Japan. Although Indian BTV-16 isolates clustered together, SRL69/Ind was segregated alone from the major cluster indicating that the isolate may not be a close relative of circulating BTV-16 viruses in India. The notion is also supported by the similar segregation of the SRL69/Ind in the ML tree, constructed based on *VP5* gene and 100% bootstrap value at the branch node where SRL69/Ind segregated separately in both the analysis.

## Conclusion

The present study indicates that the Indian BTV-16 isolates occupy a separate cluster within the Eastern topotype and show high correlation with other Indian and global BTV-16 isolates at both nucleotides and deduced aa level, whereas an isolate from Nigeria (BTV-16/NIG/AJ586694) displays a Western topotype. The data suggest that the majority of BTV-16 isolates circulating in India have a common ancestor and evolved from an ancestral Eastern topotype. The study will be helpful for selection of reference strain in serological and molecular epidemiology study in India.

## Authors’ Contributions

AS carried out the complete research work under the supervision of SKB and KC with their needful suggestion and experiment designing. Research work assisted by AC, GM, NT, and KUA and analyzed the data. MAR, JN, and ABP reviewed the manuscript and incorporated their helpful suggestions to get the manuscript better. All authors have read and approved the final manuscript.
